# Differential Levels of Alpha-2-Macroglobulin, Haptoglobin and Sero-Transferrin as Adjunct Markers for TB Diagnosis and Disease Progression in the Malnourished Tribal Population of Melghat, India

**DOI:** 10.1371/journal.pone.0133928

**Published:** 2015-08-04

**Authors:** Prachi R. Bapat, Ashish R. Satav, Aliabbas A. Husain, Seema D. Shekhawat, Anuja P. Kawle, Justin J. Chu, Hemant J. Purohit, Hatim F. Daginawala, Girdhar M. Taori, Rajpal S. Kashyap

**Affiliations:** 1 Biochemistry Research Centre, Central India Institute of Medical Sciences, Nagpur, Maharashtra, India; 2 Meditation, AIDS, Health, Addiction & Nutrition (MAHAN) Trust, C/O Mahatma Gandhi Tribal Hospital, Karmagram, Utavali, Tahsil Dharni, District: Amravati, Maharashtra, India; 3 Laboratory of Molecular RNA Virology and Antiviral Strategies, Department of Microbiology, Yong Loo Lin School of Medicine, National University Health System, National University of Singapore, Singapore; 4 Environmental Genomic Unit, National Environmental Engineering Research Institute (NEERI), CSIR, Nehru Marg, Nagpur, India; Fundació Institut d’Investigació en Ciències de la Salut Germans Trias i Pujol. Universitat Autònoma de Barcelona. CIBERES, SPAIN

## Abstract

Lack of diagnostic capacity has been a crucial barrier preventing an effective response to the challenges of malnutrition and tuberculosis (TB). Point-of-care diagnostic tests for TB in immuno-incompetent, malnourished population are thus needed to ensure rapid and accurate detection. The aim of the study was to identify potential biomarkers specific for TB infection and progression to overt disease in the malnourished population of Melghat. A prospective cohort study was conducted in the year 2009 through 2011 in six villages of the Melghat region. 275 participants consisting of malnourished cases with a) active TB (n = 32), b) latent TB infection (n = 90), c) with no clinical or bacteriological signs of active or latent TB (n = 130) and healthy control subjects (n = 23) were recruited for the study. The proteome changes of the host serum in response to *Mycobacterium tuberculosis* (*M*.*tb*) infection were investigated using one dimensional electrophoresis in combination with matrix-assisted laser desorption ionization time-of-flight mass spectrometry (MALDI-TOF MS). Three most differentially expressed proteins; alpha-2-macroglobulin (A-2-M), sero-transferrin and haptoglobin were identified by MALDI-TOF MS analysis, which were up-regulated in the malnourished patients with active TB and down-regulated in the malnourished patients compared with the healthy controls. Additionally, follow-up studies indicated that the expression of these proteins increased to nearly two folds in patients who developed active disease from latent state. Our preliminary results suggest that A-2-M, sero-transferrin and haptoglobin may be clinically relevant host biomarkers for TB diagnosis and disease progression in the malnourished population. This study provides preliminary framework for an in-depth analysis of the biomarkers in larger well-characterized cohorts. Evaluation of these biomarkers in follow-up cases may further aid in improving TB diagnosis.

## Introduction

Malnourishment is the most common cause of immunodeficiency worldwide [1, 2]. Impaired immune status is an incitement for infections leading to chronic illnesses. Malnutrition and tuberculosis (TB) are both problems of considerable magnitude in most of the underdeveloped regions of the world [3]. Although the presence of malnutrition is a recognized risk factor for the development of TB, the association of malnutrition with the extent of disease has not been well studied. Limited efforts have been made in the identification of biomarkers for accurate diagnosis of latent TB infection (LTBI) and progression to overt disease in populations where both TB and malnutrition are widespread.

**Fig 1:**
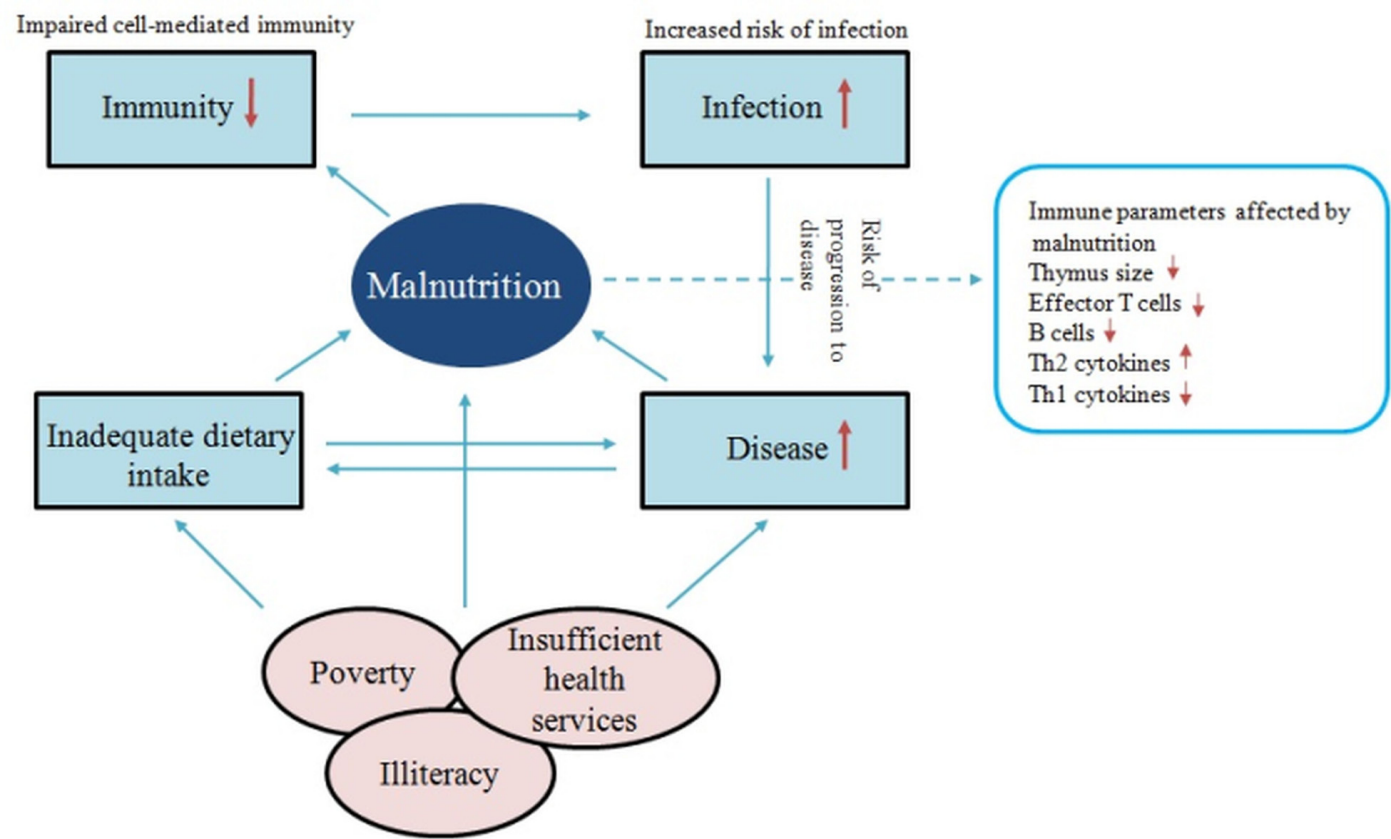
Relationship between malnutrition, infection and immunity. Malnutrition is considered the most relevant risk factor for illness and death. This direct relationship between malnutrition and death is mainly due to the resulting immunodeficiency and, consequently, greater susceptibility to infectious agents. Many factors affect the degree and distribution of malnutrition and micronutrient deficiency around the world, with poverty being the primary reason. Other factors which are also deeply involved in malnutrition include: socioeconomic instability; impaired educational development; unsanitary conditions; poor food practices and the shortage or ineffectiveness of nutrition programs.

The different manifestations of infection with *Mycobacterium tuberculosis* (*M*.*tb*) reflect the balance between the bacilli and host defense mechanisms [4]. Protective immunity to TB has been ascribed to innate as well as cell-mediated immune response [5]. However, prevalence of malnutrition, poverty and other socio-economic problems profoundly affect the immune system accounting for greater attributable risk to TB (Fig 1) [6].

The present study was conducted in the tribal belt of Melghat where malnutrition and TB are the two leading causes of morbidity and mortality [7]. Our efforts in the identification of biomarkers for active and latent TB in this population have been constant and ongoing. However, any immunomodulating factors affecting cellular immunity in vivo may impact on the test performance in vitro often yielding false negative results [8]. Despite these considerations, no immunodiagnostic test exists that can accurately diagnose active TB or distinguish asymptomatic forms of infection that are associated with a high risk of disease progression in the malnourished populations.

Given the paucity of data pertaining to TB and malnutrition, the present study aimed at elucidating potential markers for TB diagnosis and disease progression in well-defined malnourished cases from Melghat using comprehensive proteomics approach.

## Materials and Methods

### Ethics Statement

The study was approved by the Institutional Ethics Committee of the Central India Institute of Medical Sciences (CIIMS), Nagpur, and that of the Meditation Addiction Health AIDS Nutrition (MAHAN) Trust, Amravati, Maharashtra, India, and is in accordance with The Code of Ethics of the World Medical Association (Declaration of Helsinki). Written consent forms were collected from each participant after a detailed oral explanation about the study. Since the study was also conducted on children, a written informed consent was obtained from the immediate caretaker, or next of kin, prior to inclusion, on behalf of children participating in the study.

### Study Design and Participants

A prospective cohort study was conducted over a period of 2 years between May 2009 and June 2011 during the camps organized in the Melghat region of Maharashtra. This region has a high incidence of TB along with other infectious diseases. The participants for the study were recruited with the help of the MAHAN trust which provides medical care and health services to the tribal communities living in the area. According to the information procured from the trust, majority of the population shows signs of protein energy malnutrition and has nutritionally deficient dietary habits and unsafe drinking water.

For the present study, detailed information from all participants regarding age, gender, weight, height, daily dietary intake, occupation and education were recorded. Nutritional assessment of the participants was done by dieticians through standardized questionnaires (S1 File) based on parameters described elsewhere [9]. In addition, each participant was also asked to complete a questionnaire about his/her possible risk of exposure to *M*. *tb*, type and duration of exposure with active TB patients, details of any prior Tuberculin skin test (TST), the presence of underlying illnesses, infections experienced in the last 3 months, medication, history of previous TB or anti-TB treatment (ATT). The Bacillus Calmette—Guérin (BCG) vaccination status was assessed based on examination of the BCG scar on left forearm.

**Fig 2:**
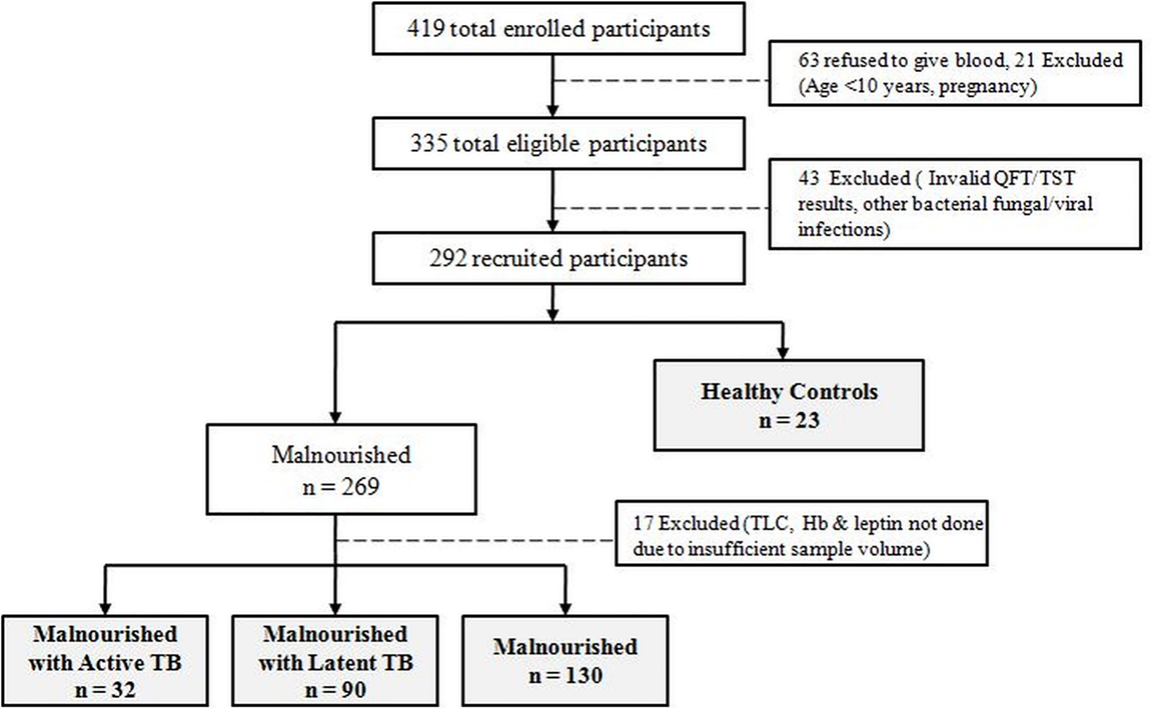
Study participation diagram. The figure represents the inclusion/exclusion criteria adopted for recruitment of the study population. The population was categorized into four groups namely: Malnourished group with Active TB (n = 32), Malnourished group with Latent TB (n = 90), Malnourished group (n = 130) and Healthy control group (n = 23) (Grey boxes indicate the groups included in the final analysis). TB-Tuberculosis, TST-Tuberculin Skin Test, Cut-off point is atleast 10 mm, QFT- QuantiFERON-TB Gold, Cut-off is atleast 0.35 IU/μl. TLC-Total leukocyte count, Hb-Hemoglobin count (g/ml)

A total of 419 participants were enrolled for the study. Of these individuals, a total of 63 participants refused to give blood and were excluded from the study. Pregnant women (n = 12) and children below the age of 10 years (n = 9) were also excluded to avoid the impact of BCG reactivity on TST. 14 participants with invalid TST or QuantiFERON-TB Gold test (QFT) results and 29 participants who had other bacterial/fungal or viral infections were also excluded. The remaining 292 participants were recruited for further analysis. A minimum sample size of 267 participants was calculated. Fig 2 represents the inclusion/exclusion criteria adopted for recruitment of the study population.

#### Control group (n = 23)

The participants in this group had no clinical, bacteriological features of TB or any other diseases, had normal chest radiographs and had no history of ATT. These individuals also had negative TST/QFT test results with no past or recent exposure to active TB cases. This group consisted of individuals only with body mass index (BMI) values ranging from 18 to 24.

#### Criteria for Malnourishment

All the recruited participants underwent nutritional assessment and physical examination. Patients were weighed and their heights measured, whenever clinically possible, using the scale available. Percentage of weight loss and BMI were calculated. Screening for malnutrition involved assessment of BMI and nutrient intake. In addition, serum determinations of leptin, albumin, globulin, total protein, total lymphocyte count (TLC), etc were also done. Based on the nutritional assessment, a total of 269 malnourished participants were selected; out of which 17 participants whose hematological/biochemical parameters could not be evaluated due to insufficient sample volume were eliminated from the study.

#### Malnourished group with Active TB (n = 32)

Malnourished participants with clinical course consistent with active TB (fever, cough, suggestive X-ray) and sputum smear positive for *M*.*tb* culture/ AFB bacilli were included in the present group.

#### Malnourished group with Latent TB (n = 90)

This group consisted of individuals having either a positive QFT/TST result with no evidence of active disease, negative chest radiograph and no previous history of treatment. These participants were close contacts of active TB cases with an exposure period of more than 3 months.

#### Malnourished group (n = 130)

This group consisted of malnourished individuals without TB (active or latent) or any other secondary illnesses.

#### Sample collection

5 ml of venous blood was collected from all participants for the QFT assay. Additionally, 5 ml of venous blood was collected and allowed to clot. Serum was separated after centrifugation at 12000 r.p.m. The samples were stored at -20°C until they were used. One aliquot of the samples was used to perform a complete blood count and evaluation of other biochemical parameters. The other aliquot of samples were subjected to one dimensional sodium dodecyl sulphate—polyacrylamide gel electrophoresis (SDS-PAGE).

#### Tuberculin Skin Test (TST) and QuantiFERON-TB Gold test (QFT)

The TST was performed by the Mantoux method using 5 tuberculin units (T.U.) of purified protein derivative (PPD) (Span Diagnostics, India). The TST was administered intradermally by a certified technician and read after 48 to 72 hours. In accordance with the Revised National Tuberculosis Control Programme, an induration of at least 10 mm was considered positive. Prior to performing the TST, 5 mL of venous blood was drawn into two heparin-containing tubes to perform the QFT assay. The QFT was performed according to the manufacturer’s instructions and 0.35 IU/mL was selected as the cut-off value.

#### Hematological and Biochemical parameters

Blood serum from the study participants was used to perform a complete blood count including red blood cell count, total leukocyte count, platelet and hemoglobin (Hb) count. Additionally, biochemical parameters like fasting glucose, total protein, albumin and globulin were also evaluated in the serum. All measurements were performed in the Pathology department, CIIMS, Nagpur using commercial kits according to the manufacturer's recommendations.

Serum Leptin levels were estimated using SPIbio Human EIA kit. This Enzyme Immunometric Assay (EIA) is based on the double antibody sandwich technique. In brief, serum samples were diluted in the EIA buffer and added in the pre-coated 96 well plate with anti—Leptin antibody. The plate was then incubated for an hour and washed thrice with the wash buffer. This was followed by addition of the anti-Leptin tracer to the wells which was again incubated and washed thrice as above. Substrate solution was dispensed onto the plate, which was incubated in dark and excess colour development was stopped by adding stop solution. The absorbance was read at 450 nm by a colorimeter. Concentration of leptin in the serum samples was calculated by comparing the O.D. value of samples with the O.D. of standard leptin concentrations.

#### One-dimensional SDS PAGE

Electrophoresis was performed with vertical slab gel electrophoresis system (Biotech, Yercaud) by standard Laemmlli’s method using 10% running gel and 5% stacking gel. Electrophoresis was carried out at 200 Volts. Serum sample volume corresponding to 40 μg of protein concentration was added per well. Gels were developed by staining with Coomassie brilliant blue R-250 and the protein profiles were studied by densitometric analysis. Band size (i.e., molecular weight) was estimated using molecular weight markers (Genei, Bangalore, India).

#### Image Analysis

Image analysis was done using the Image Lab software 4.0 (Bio-rad Laboratories) to create a reproducible and automated workflow for imaging and analyzing gels. The relative quantity tool was used to create a comparative profile of control and test samples which was represented in terms of pixel intensity. Using the auto analysis setting three protein bands of molecular weight 165 kDa, 79 kDa and 47 kDa were identified as the most differentially expressed bands. These prominent bands of the one-dimensional gel were then cut and subjected to in-gel digestion.

#### In-gel digestion and MALDI-TOF MS

The in-gel digestion and MALDI-TOF MS of each excised protein bands were kindly performed by Protein and Proteomics Centre, National University of Singapore (Singapore). The proteins were identified by searching in the National Centre for Biotechnology Information non-redundant (NCBInr) database using a MASCOT program (http://www.matrixscience.com). No thresholding was applied to the MS/MS fragment ions intensities.

#### Follow-up study

For follow up studies, participants were recruited based on the information from earlier records available with us and also with the help of the MAHAN Trust. Based on brief counseling; we were able to recruit 9 participants. The recruited participants were screened for existing panel of TB biomarkers reported earlier in our laboratory using protocols described elsewhere [10–14]. Based on the clinical course of TB infection, participants were categorized into three distinct groups. Category I consisted of individuals who had LTBI and developed active disease. (n = 1). Category II comprised of participants who remained latently infected, showing no evidence of active disease after follow-up analysis (n = 5). Category III consisted of individuals who initially had active TB and continued to show symptoms of TB after follow-up analysis (n = 3).

#### Statistical Analysis

The sample size calculations were done using Raosoft software (Raosoft, Inc.). The frequencies (percentage) of the demographic characteristics were measured on a nominal scale. ANOVA test was carried out to test the significance of difference in the mean levels of hematological parameters for the four study groups. Categorical variables were described as mean ± standard deviation (SD). The level of significance was established for a probability of 0.05 based on the Tukey correction. The association between the baseline parameters and the study groups was measured using the Χ^2^ Test and the agreement was calculated by contingency tables using the contingency co-efficient. The level of significance was established for a probability of 0.05. All tests were performed using GraphPad Prism (version 5.03) and MedCalc statistical software (version 10.1.2.0). Graphs were plotted using GraphPad Prism (version 5.03). Error bars represent mean ± standard deviation. Pie charts were drawn using Microsoft Office Excel 2007. Comparison of TB biomarkers in follow-up cases was also performed using Microsoft Office Excel charts with conditional formatting.

## Results

Out of the 419 participants that were enrolled, a total of 275 participants who met the eligibility criteria were included in the study for analysis. Sample description is presented in Table 1. Among the 275 participants, 156 (56.7%) were males and 119 (43.3%) were females. Nearly 161 participants (58.6%) belonged to the 18–40 years age group. 252 (91.6%) participants belonged to the underweight category with BMI lower than 18.5. Individual nutritional assessment indicated that as high as 193 (70.2%) participants had poor dietary intake and 85 (33.7%) were severely malnourished. The BCG vaccination status was confirmed only in 82 (29.8%) participants. 188 (68.4%) out of the total 275 participants belonged to the lower socioeconomic group based on the *Kuppuswamy* rating system applicable to Indian families.

**Table 1:** Demographic characteristics of the population under study.

Characteristics	Level	Total number (Percentage)
Age (years)	< 18	38 (13.8)
	18–40	161 (58.6)
	> 40	76 (27.6)
Gender	Male	156 (56.7)
	Female	119 (43.3)
BMI	> 18.5	252 (91.6)
	18.5–24	23 (8.4)
	> 24	0 (0.0)
Malnourishment†	Moderate	167 (66.3)
	Severe	85 (33.7)
BCG Vaccination	Yes	82 (29.8)
	No	193 (70.2)
Socioeconomic status	Low	188 (68.4)
	Better	87 (31.6)
Dietary Habits	Poor	193 (70.2)
	Better	82 (29.8)

Characteristics of 275 subjects from Melghat are listed in the table. Percentages in different categories are indicated in parentheses. BMI- Body mass index categorization recommended by World Health Organization (WHO). Severity of malnourishment was based on parameters assessed through nutritional questionnaires. The socio-economic status was decided based on the *Kuppuswamy* rating system applicable to Indian families.

^**†**^ Out of 252 subjects

### Evaluation of hematological parameters

Table 2 presents the comparison of hematological parameters in the four study groups. High blood glucose levels were seen in the malnourished group with active TB compared to the other study groups (P < 0.0001). Total serum protein, albumin, hemoglobin (Hb), red blood cell (RBC) count and lymphocyte count (L %) were found to be significantly lower in the malnourished group with active TB compared to the healthy control group. In contrast, the platelet count, total leukocyte count, polymorphnuclear neutrophils (P %), eosinophils (E %) and monocyte count (M %) were significantly elevated in the malnourished group with active TB. The total blood cell count barring the RBC count was significantly higher in the malnourished group with active TB compared to the malnourished group with latent TB. The malnourished group was associated with lower levels of glucose, protein, albumin, RBC count, platelet count, L% and Hb as against the healthy control group (P < 0.0001).

**Table 2:** Hematological parameters of the population under study.

Hematological parameters	Malnourished with Active TB	Malnourished with Latent TB	Malnourished	Healthy Control	p value
	n = 32	n = 90	n = 130	n = 23	(ANOVA)
Blood glucose (72 to 110 mg/dL)	79.36 ± 2.66	58.76 ± 3.63	53.99 ± 3.87	72.02 ± 2.45	P < 0.0001 *
Total protein (6.4–8.3 g/dL)	6.2 ± 1.5	6.53 ± 0.39	6.24 ± 0.83	7.84 ± 1.1	P < 0.0001 *
Albumin (3.5–5.0 g/dL)	3.50 ± 0.27	3.81 ± 0.45	3.4 ± 0.21	4.13 ± 1.8	P < 0.0001 *
Globulin (2.3–3.5 g/dl)	3.41 ± 0.55	3.33 ± 0.62	4.23 ± 0.57	3.42 ± 0.22	P < 0.0001 *
RBCs (4.3–6.2 x 10^6^/cumm)	4.73 ± 0.67	4.95 ± 0.55	4.81 ± 0.56	5.49 ± 0.35	P = 0.9999
Platelets (150–400 x 10^3^/cumm)	291.8 ± 68.9	227.43 ± 61.56	224.7 ± 72.06	243.4 ± 63.59	P < 0.0001 *
TLC/cumm (4000–11000/cumm)	8800 ± 3013	8490 ± 2509	8353 ± 1844	7853 ± 1653	P = 0.4539
P % (35–80%)	72.18 ± 2.43	69.32 ± 1.98	66.11 ± 2.57	61.98 ± 0.43	P < 0.0001 *
L % (40–75%)	32.15 ± 1.78	31.45 ± 2.1	28.97 ± 0.12	43.81 ± 2.32	P < 0.0001 *
E % (1–6%)	7.28 ± 0.67	5.58 ± 1.56	4.08 ± 1.26	3.5 ± 1.43	P < 0.0001 *
M % (2–10%)	3.4 ± 2.6	2.89 ± 0.98	2.15 ± 1.57	1.55 ± 0.58	P < 0.0001 *
Hb (g/dl) (14–18)	10.88 ± 1.5	11.07 ± 1.95	10.1 ± 2.1	14.20 ± 1.52	P < 0.0001 *

Hematological parameters of 275 subjects from Melghat are listed in the table. Complete blood count was done in the Pathology department, CIIMS, Nagpur using commercial kits. The normal detection ranges of each parameter are indicated in the brackets. P < 0.05 was considered statistically significant. Values are reported as Mean ± SD. Statistical analysis was done using GraphPad Prism (version 5.03). Statistical variance between groups was calculated using mean values through ANOVA test with Tukey post test.

* Statistically significant when the significance level is set as P < 0.05 based on the Tukey correction

### Baseline characteristics

Baseline characteristics of the study population are indicated in Table 3. Categorical variables are described as the number of cases and percentages. Assessment of baseline characteristics in the study groups indicated 83% TST positivity and 59% QFT positivity in the malnourished group with latent TB as against 41% TST and QFT positivity each in the malnourished group with active TB. In accordance with the inclusion criteria, the highest positive responders for clinical parameters like weight loss, cough with and without expectorant, abdominal pain and chest pain were seen in the malnourished group with active TB. (Fever 88%, P < 0.0001; Weight loss 72%; P < 0.0001; Cough with expectorant 47%, P = 0.0002; Cough without expectorant 72%, P = 0.0001; Abdominal pain 22%, P = 0.0001; Chest pain 47%, P = 0.0001). The relationship between risk factors like smoking, tobacco chewing, alcohol consumption and the study groups was also assessed. No significant association between smoking and alcohol consumption with any of the study groups was observed (Χ^2^ = 3.103, P = 0.3759; Χ^2^ = 1.786, P = 0.6179 respectively). 34% tobacco chewers belonged to the malnourished group with active TB however the association was not very statistically significant (Χ^2^ = 9.331, P = 0.0252).

**Table 3:** Baseline characteristics of population under study.

Baseline characteristics	Level	Malnourished with Active TB	Malnourished with Latent TB	Malnourished	Healthy Control	Χ^2^	Df	p value	Contingency Coefficient
		n = 32	n = 90	n = 130	n = 23				
QFT	Positive	13 (41)	53 (59)	0 (0)	0 (0)	113.226	3	P < 0.0001	0.54
TST	Positive	13 (41)	75 (83)	0 (0)	0 (0)	182.083	3	P < 0.0001	0.631
Fever	Yes	28 (88)	21 (23)	26 (20)	0 (0)	71.317	3	P < 0.0001	0.454
Weight Loss	Yes	23 (72)	17 (19)	26 (20)	0 (0)	49.903	3	P < 0.0001	0.392
Cough with Expectoration	Yes	15 (47)	28 (31)	25 (19)	0 (0)	20.069	3	P = 0.0002	0.261
Cough without expectoration	Yes	23 (72)	16 (18)	35 (27)	0 (0)	45.18	3	P = 0.0001	0.376
Abdominal pain	Yes	7 (22)	4 (4)	0 (0)	0 (0)	33.048	3	P = 0.0001	0.328
Chest pain	Yes	15 (47)	8 (9)	0 (0)	0 (0)	75.683	3	P = 0.0001	0.465
Smoking	Yes	3 (9)	11 (12)	13 (10)	0 (0)	3.103	3	P = 0.3759	0.106
Tobacco	Yes	11 (34)	23 (26)	34 (26)	0 (0)	9.331	3	P = 0.0252	0.181
Alcohol	Yes	4 (13)	17 (19)	22 (17)	2 (9)	1.786	3	P = 0.6179	0.08

Characteristics of 275 subjects with the number of positive responders for each category are presented in the table. Percentages are indicated in parentheses. The statistical variance between groups was calculated using Chi squared test and p value. P < 0.05 was considered statistically significant. Statistical analysis was done using MedCalc statistical software (version 10.1.2.0).

Df, Degrees of freedom.

### Mean BMI, Leptin, QFT and TST values in the different study groups

**Fig 3:**
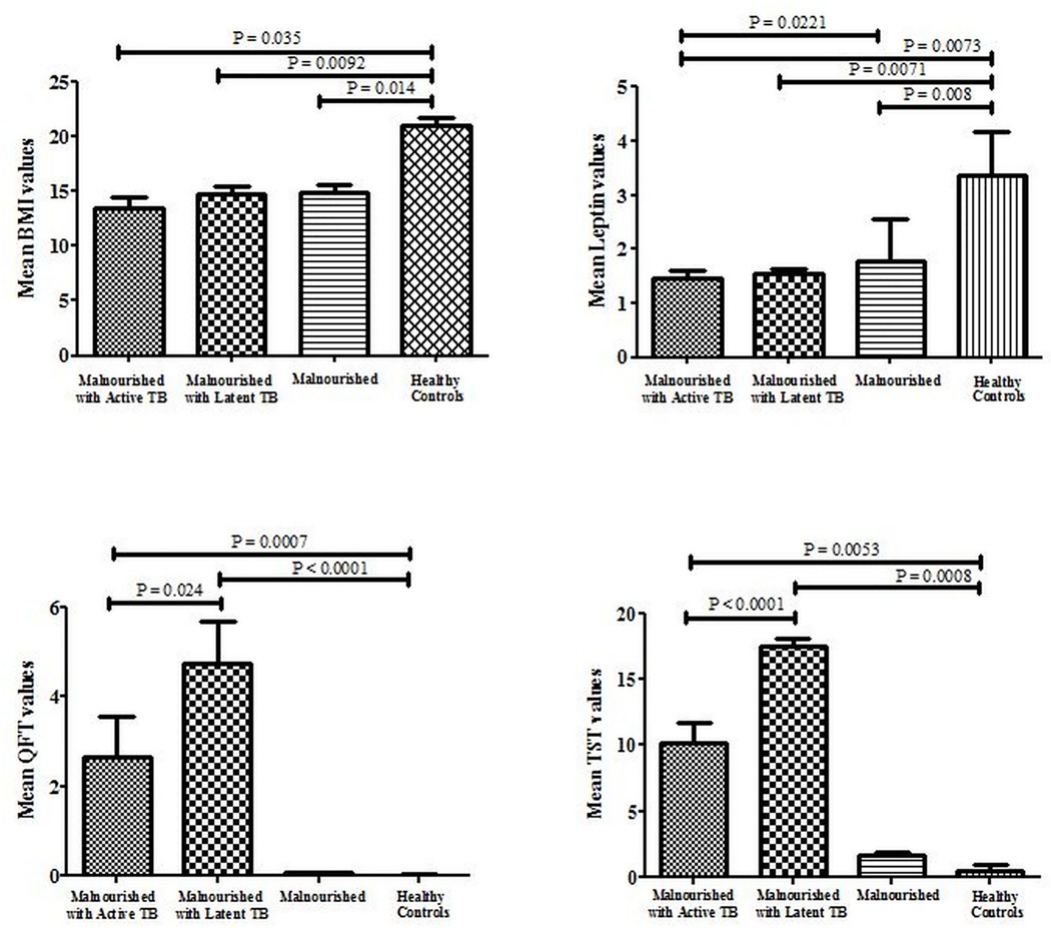
Mean BMI, Leptin, QFT and TST values in different categories. Bar graphs represent the mean values in different study groups. Graphs were plotted using GraphPad Prism (version 5.03). Error bars represent mean ± standard deviation. BMI- Body mass index, TST-Tuberculin Skin Test, Cut-off point is atleast 10 mm, QFT- QuantiFERON-TB Gold, Cut-off is atleast 0.35 IU/μl, Leptin values are indicated as absorbance at 450 nm. Leptin levels were evaluated in serum SPIbio Human EIA kit.

Fig 3 shows the comparison of mean BMI, Leptin, QFT and TST values in the different study groups. The mean BMI values were significantly higher in the healthy control group compared to the other groups (P < 0.05). Leptin, a marker of energy metabolism was found to be significantly down-regulated in the malnourished group with active TB compared to the other groups (P < 0.05). QFT results indicated that the malnourished group with latent TB had the highest QFT values (indicated in terms of U/ml of IFN-γ secretion). The malnourished group with active TB also showed significantly high mean QFT values however these were comparatively lower than the malnourished group with latent TB (P < 0.05). The mean TST values (expressed in terms of induration in mm) were also significantly higher in the malnourished group with latent TB compared to the other study groups (P < 0.0005).

### Image Analysis and MASCOT results

**Fig 4:**
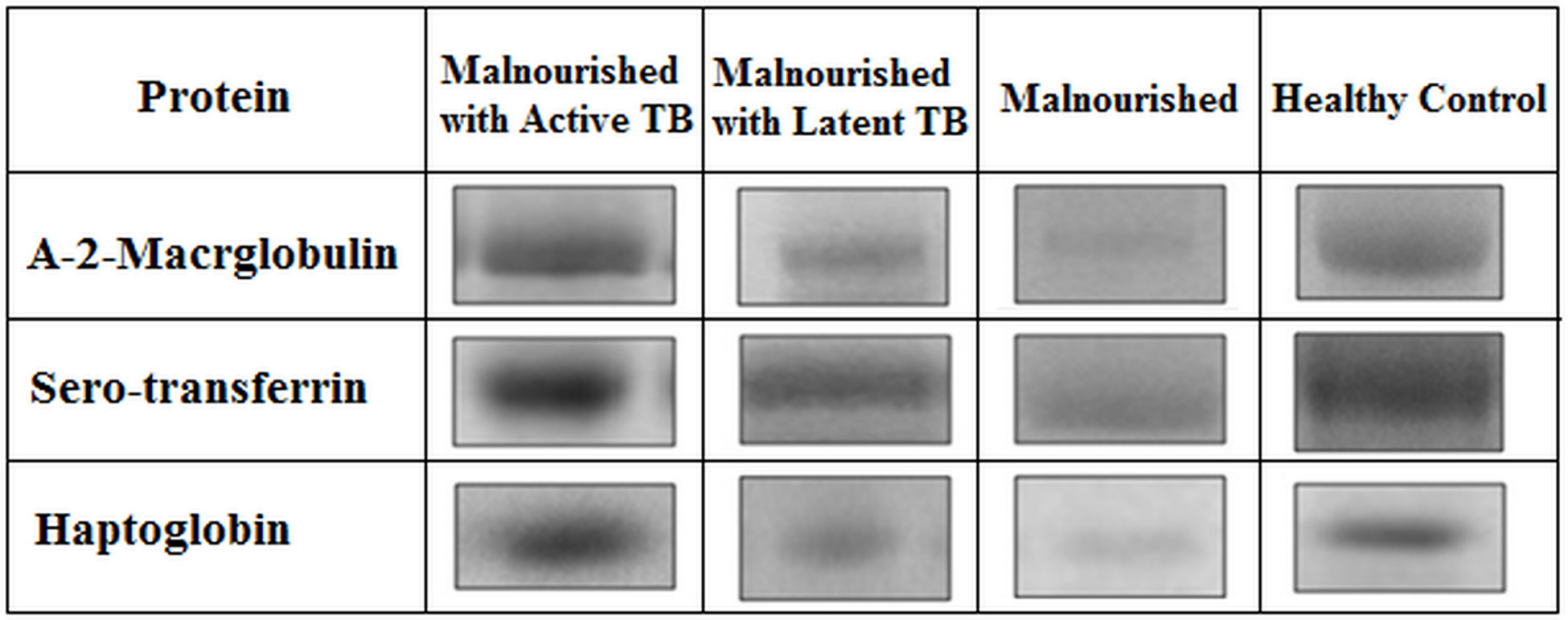
Representative 1-DE gel images of identified proteins in different categories. Boxes indicate the differentially expressed protein bands in different study groups. Gel imaging and analysis was done using Image Lab software (version 4.0, BioRad). The protein bands in the test groups were compared against healthy control group.

Three differentially expressed protein bands with molecular weights 165 kDa, 79 kDa and 47 kDa were identified by SDS PAGE analysis. Representative montage images for these differentially expressed proteins are shown in Fig 4. The intensities of the three protein bands were highest in the malnourished group with active TB compared to the other groups. However there was a significant down-regulation of protein expression in malnourished cases compared to the healthy control subjects.

### Densitometric analysis

**Fig 5:**
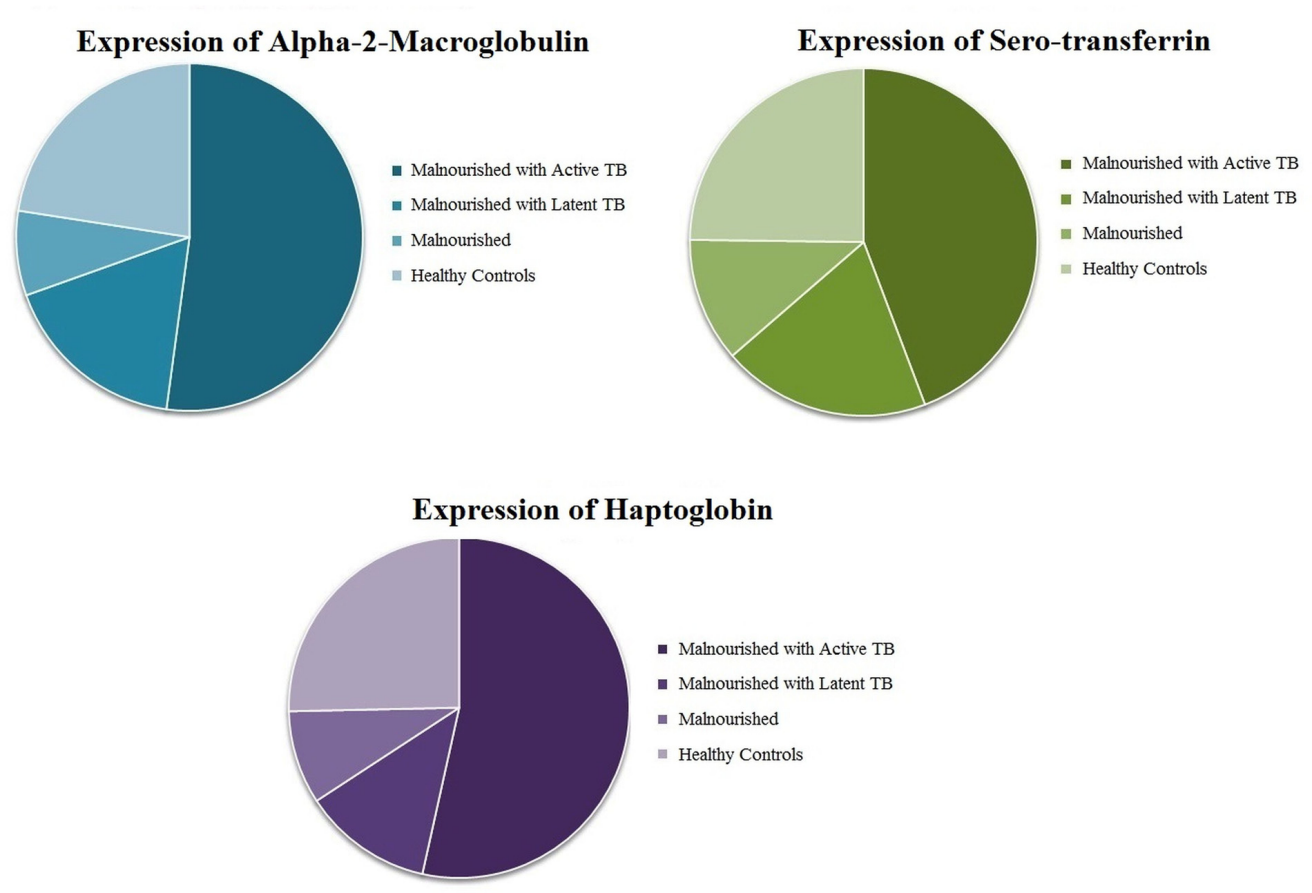
Expression of proteins by densitometric analysis. Pie charts represent the intensities of protein bands assessed by densitometric analysis in different study groups. Gel analysis was done using Image Lab software (BioRad) (Version 4.0)

Densitometric analysis of the most differentially expressed proteins identified that the levels of the three proteins in the malnourished with active TB samples were increased on an average by 1.8 fold to 2.3 fold compared to the healthy control samples. In contrast, the levels of these proteins in the malnourished samples decreased on an average by 2.1 to 2.9 fold. The protein levels in the malnourished with latent TB cases were also found to be down-regulated on an average by 2.3 to 4.3 fold compared to the malnourished group with active TB. Fig 5 presents the densitometric analysis of the differentially expressed proteins in the four study groups.

### Bioinformatics

The results of MALDI-TOF MS analysis are summarized in Table 4. Table also enlists the protein mass and score. Based on the mass spectrometric results and bioinformatics analysis the three differentially expressed proteins were identified as Alpha-2-macroglobulin (A-2-M), Sero-transferrin and Haptoglobin. A-2-M and sero-transferrin showed 35 peptide sequence matches with 21% and 35% coverage. Haptoglobin showed 16 peptide query matches with sequence coverage of 24%.

**Table 4:** Mascot Search Results.

Protein identified	Accession no.	Mr	Score	Peptide matches	Coverage
Alpha-2-Macroglobulin (A-2-M)	gi|308153640	164613	676	35	21%
Sero-transferrin (Transferrin)	gi|313104271	79294	691	35	35%
Haptoglobin	IPI00641737	47378	485	16	24%

The peptide mass list from MS analysis was used to search the NCBI database using the MASCOT search engine (Matrix Science). The taxonomy used was human or bacteria. Table lists the proteins identified by MS analysis together with details of individual mass value matches.

Mr, Relative molecular mass

### Evaluation of TB biomarkers in follow-up cases

**Fig 6:**
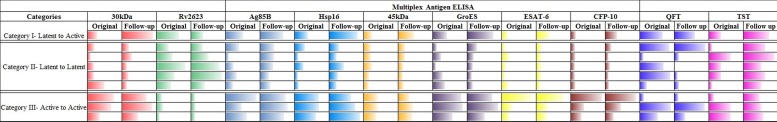
Evaluation of TB biomarkers in original and follow-up samples of the population under study. Chart shows a comparison between the levels of TB biomarkers (30 kDa antigen, ADA; Adenosine deaminase, Mycobacterial Dormancy Regulon Protein Rv2623, Multiplex antigens; Ag85B; *M*.*tb* Ag85B secretory protein, Hsp-16; 16 kDa heat-shock protein, 45kDa antigen, GroES; *M*.*tb* 10kDa chaperonin, ESAT-6; 6 kDa early secretory antigenic target and CFP-10; 10 kDa culture filtrate protein, QFT; QunatiFERON-TB Gold test and TST; Tuberculin Skin Test) in original and follow-up cases of Melghat categorized into three groups; Latent to Active TB group (n = 1), Latent to Latent TB Group (n = 5) and Active to Active TB group (n = 3).

Follow-up studies were carried out to associate the expression of proteomic biomarkers with existing markers of TB (Fig 6). The results revealed that the individuals who went on to develop active disease from latent stage (Category I) showed significantly elevated antigen (30 kDa) and antibody levels (Ag85B, Hsp-16, 45kDa, GroES, ESAT-6 and CFP-10) as well as increased QFT and TST values. The titers of *M*.*tb* antigen/antibody were significantly lower in individuals with latent infection (Category II) as compared to those observed in active TB cases. In contrast, the Rv2623 titers were found to be significantly raised in both original and follow-up cases of latently infected individuals. Individuals with Active TB (Category III) showed considerably increased antigen and antibody titers and decreased Rv2623 titers.

## Discussion

Nutritional status of the host defines the ability of the immune system to prevent infection and disease. Various reports are in common agreement that malnutrition is associated with immune deficiency and increases susceptibility to infection [15–17]. The association of malnutrition and TB is also well recognized, as micro and macro deficiency impaired immune response increases the risk of TB among malnourished individuals. These nutrient deficiencies are also known to cause immunosuppression and dysregulation of the immune responses. Accurate detection of both latent and active form of TB thus constitutes an important part of health management programs in order to reduce the burden of TB in such populations. The present study was an attempt to identify biomarkers of TB disease and disease progression in the malnourished population of Melghat. All the participants recruited for this study belonged to the Korku tribe which is dominant in this region. Based on the information procured from the MAHAN trust, majority of the population here shows signs of protein energy malnutrition (> 90%) [18]. In the present study, to ascertain the health status of the population we analysed known parameters of malnutrition including dietary intake, BMI, leptin and serum determinations of albumin, globulin, total protein, TLC etc. Based on our nutritional questionnaires we observed that 70% of the population had poor dietary intake and 33.7% of the population was severely malnourished. The obtained BMI values for the population also supported the categorization of the selected population as malnourished. For further confirmation, we also evaluated leptin in the collected serum samples. Leptin, a 16 kDa protein product of the obese gene has been proposed to act as a link between nutritional status and immune function [19–22]. We observed decreased level of leptin in the malnourished group compared to the healthy controls. The malnourished with active TB group however showed the most down-regulated leptin levels. Our results are consistent with the study done by Wieland CW *et al* wherein leptin-deficient mice had a moderately impaired immune response leading to increased susceptibility to pulmonary infection with *M*.*tb* [22]. In yet another study, decreased levels of leptin were observed in mice with prolonged fasting and malnutrition [23]. Evaluation of hematological parameters indicated that the malnourished group was associated with lower levels of glucose, protein, albumin, RBC count, platelet count, L% and Hb when compared to the healthy controls. Low albumin and RBC count have been regarded as constant features of malnutrition. Several reports have suggested that the lowered RBC count may be an adaptation to low metabolic oxygen requirements and decrease in lean body mass. Low L% in the malnourished individuals can be attributed to the thymic atrophy correlated with depletion of lymphocytes causing impaired immunity [24, 25]. In contrast, the malnourished group with active TB was associated with high TLC and P% which are hallmarks of acute inflammation.

Proteomics has emerged as a powerful approach for identifying disease biomarkers. The technique has been intensively studied in TB disease and several promising candidates have been developed to diagnose TB in various populations [13, 26, 27]. However, the existing biomarkers appear to be inefficient in diagnosing TB in malnourished individuals due to their impaired immune status [28, 29]. In our study, proteome analysis of malnourished individuals with a) active TB, b) latent TB infection, c) with no clinical or bacteriological signs of active or latent TB and healthy controls identified three most differentially expressed proteins namely; Alpha 2-Macroglobulin (A-2-M), Sero-transferrin and Haptoglobin in the serum samples from the four study groups. These proteins were found to be up-regulated in the malnourished group with active TB as compared to the other study groups and significantly down-regulated in malnourished group as compared to the healthy controls.

A-2-M and Haptoglobin are acute phase proteins (APPs) that have been previously reported in the serum samples of TB patients [30–32]. A-2-M is a well known carrier protein that binds to numerous growth factors and cytokines such as TNF-α, IL-6, IL-1β etc. These cytokines have a crucial role in the defense against *M*.*tb* infection, thereby suggestive of the functional significance of A-2-M in TB disease [33, 34]. Haptoglobin is the primary hemoglobin (Hb) binding protein in human plasma, which attenuates the adverse biochemical and physiologic effects of extracellular Hb [35, 36]. Another APP identified by MS analysis was sero-transferrin which is an iron-binding plasma glycoprotein that controls the level of free iron in biological fluids [37]. In cellular models it has been reported that excess iron markedly increases mycobacterial growth [38, 39]. Thus the observed increase in sero-transferrin and haptoglobin in the active TB patients may indicate the extent of *M*.*tb* infection in these patients. Although non-specific; APPs have been proposed as candidate biomarkers for active TB in various studies [40–42]. However, none of the studies were reported in malnourished TB populations. In the present study, we report APPs as important biomarkers in the malnourished TB population of the Melghat region. Interestingly, an increase in all the three identified proteins was observed in participants who progressed to active TB from latent infection indicating their dual role as disease progression markers in the malnourished population. Table 5 shows the functional classification of proteins identified by MS analysis.

**Table 5:** Protein functions in tuberculosis and malnutrition.

Protein	Function	References
Alpha-2-Macroglobulin	Protease inhibitor and cytokine transporter. Alpha-2-macroglobulin levels have been found to be significantly raised during tuberculosis infection. Acts as a leptin binding protein that influences the bioavailability of leptin in human plasma.	Birkenmeier G. et al, 1998; Adedapo KS et al, 2009; Kumar PN et al, 2013
Sero-Transferrin	Iron-binding blood plasma glycoproteins that control the level of free iron in biological fluids. Decreased level of transferrin causes protein energy malnutrition (Kwashiorkar, Marasmus). Levels of transferrin are reduced in newly diagnosed PTB patients.	Le Banh et al, 2006; Fu YR et al, 2012; Jain S et al, 2011
Haptoglobin	Binds free hemoglobin released from erythrocytes with high affinity and thereby inhibits its oxidative activity. Decreased level of Haptoglobin causes decrease in Red blood cell count and decreased amount of hemoglobin. Levels of haptoglobin are reduced in newly diagnosed PTB patients.	John FM et al, 1998; Quaye IK et al, 2008; Adedapo KS et al, 2009

Table enlists the roles of the three identified proteins in malnutrition and tuberculosis reported in various studies.

The acute phase response (APR) is a prominent systemic reaction of the organism to local or systemic disturbances in its homeostasis caused by infection, tissue injury, trauma or surgery, neoplastic growth or immunological disorders [43, 44]. Infection and malnutrition have a synergistic relationship. It has been reported that malnutrition leads to negatively changed hepatic synthesis which ultimately results in impaired APP response [45]. Thus, it is quite evident that the identified APPs including A-2-M, haptoglobin and sero-transferrin have critical significance in malnourished individuals. However, the role of APPs in malnourished TB population is complex and needs to be elucidated for better understanding of the disease. Analysis in follow-up cases established a two fold increase in the level of the three proteins in individuals who progressed from latent to active TB. To further confirm the specificity of these identified proteins in TB disease progression and sero-conversion, we correlated them with the available panel of mycobacterial antigens and antibodies earlier established in our laboratory [10–14]. The results demonstrated a positive correlation between APPs and TB-specific biomarkers, for identification of latency and progression to active disease.

**Fig 7:**
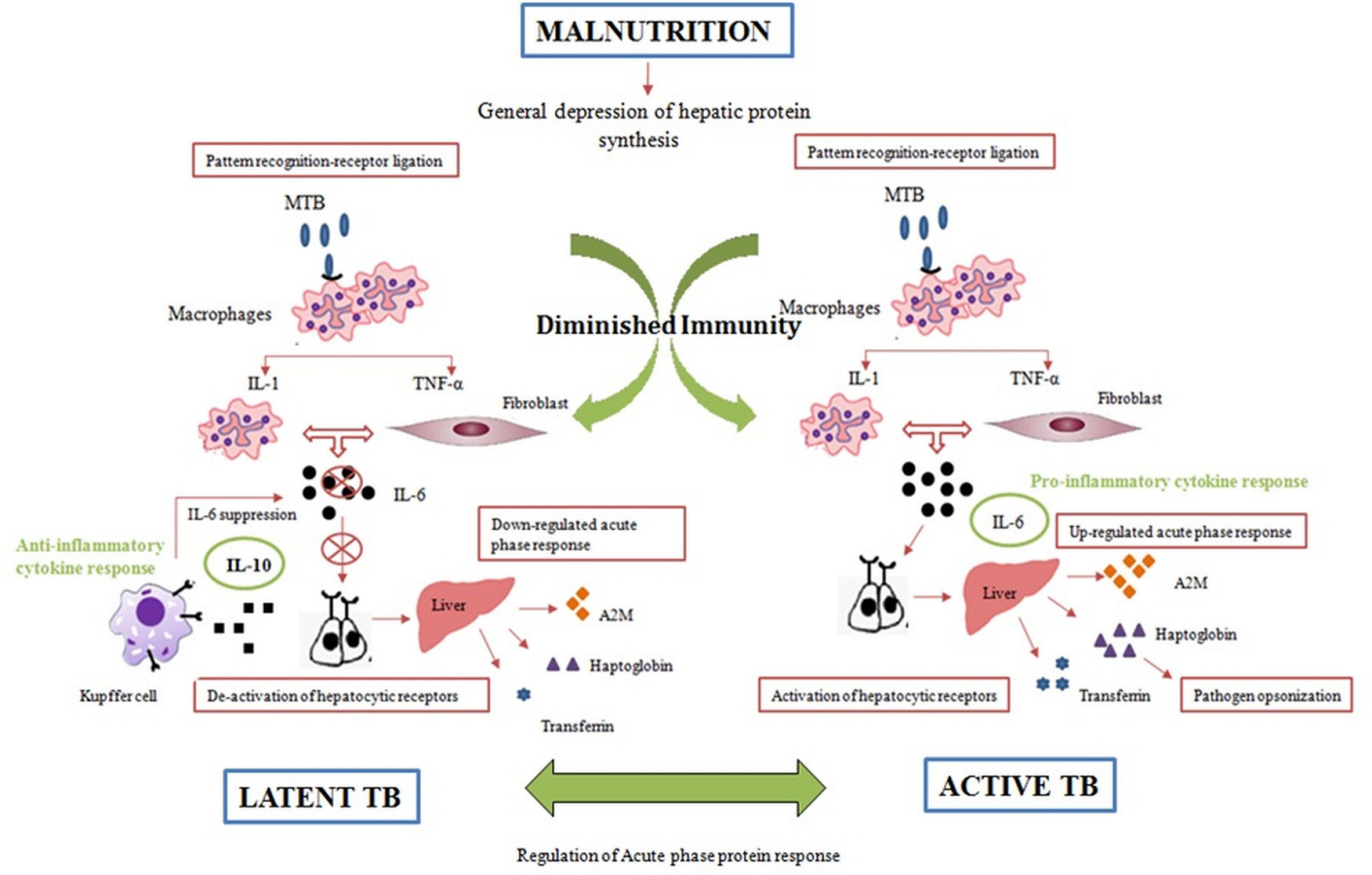
Mechanisms involved in the acute phase protein response in malnutrition and TB. Malnutrition and the anorectic effects of pro-inflammatory cytokines in the brain result in a negatively changed hepatic synthesis. In diseased condition, the pro-inflammatory cytokines TNF-α, IL-1 and IL-6 play a key role in the hepatic APR. They activate hepatocytic receptors, and synthesis of varying APPs starts. During latent form of infection, release of IL-10 by the Kupffer cells results in suppression of the local IL-6 production by gene suppression pathways coactivated on receptor binding. This results in rapid hepatic removal of circulating cytokines followed by down-regulation of hepatocytic APR.

During TB disease, several cytokines are released as an immune response against *M*.*tb* and IL-6 plays a critical role in limiting the infection. IL-6 is the major mediator for the hepatocytic secretion of APPs [46, 47]. However, during latent infection decreased levels of IL-6 and elevated levels of IL-10 have been reported which may be the reason for the lower expression of APPs in latently infected individuals [48]. Some studies suggest that release of IL-10 by the Kupffer cells results in suppression of the local IL-6 production by gene suppression pathways coactivated on receptor binding. This results in rapid hepatic removal of circulating cytokines followed by down-regulation of hepatocytic APR [43] (Fig 7). In the presented evidences a hypothesis can be generated that as the disease progresses from latent to active stage the secretion of IL-6 increases which results in the gradual increase of APPs as an immune response against *M*.*tb*. An increase in the three APPs in participants who progressed from latent to active TB supports our generated hypothesis.

The study provides significant information about the possible biomarkers for TB in the malnourished population however suffers from small sample size. Despite intensive efforts we were unable to collect significant number of follow-up samples because of the negligence of the tribal population towards medical issues. Secondly, given the broad spectrum of functional significance of APPs, inclusion of an additional group for infection control would be more comprehensible. Our observations thus require investigation in other infectious groups to further confirm the study outcome.

In conclusion, the need for TB biomarkers in the malnourished population arises due to the difficulty of accurately diagnosing TB infection. Proteomics offers the opportunity to develop new biomarkers for early and accurate diagnosis. The identified proteins A-2-M, Haptoglobin and Sero-transferrin may serve as important adjunct biomarkers for diagnosis of TB in malnourished population. These identified biomarkers also play a crucial role in TB progression. The study opens new avenues for further testing of the identified markers at the clinical point-of-care which may lead to an inexpensive and valuable test to assist clinicians in diagnosing TB in the malnourished population.

## Supporting Information

S1 File Questionnaire for nutritional assessment. (PDF)

## References

[1] Katona P, Katona-Apte J. The interaction between nutrition and infection. Clin Infect Dis. 2008 5 15; 46(10):1582–8. 10.1086/587658 18419494

[2] Chandra RK. Nutrition and the immune system: an introduction. Am J Clin Nutr. 1997 8; 66(2):460S–463S. 9250133 10.1093/ajcn/66.2.460S

[3] Gupta KB, Gupta R, Atreja A, Verma M, Vishvkarma S. Tuberculosis and nutrition. Lung India. 2009 1; 26(1):9–16. 10.4103/0970-2113.45198 20165588 PMC2813110

[4] Raja A. Immunology of tuberculosis. Indian J Med Res. 2004 10; 120(4):213–32. Review. 15520479

[5] van Crevel R, Ottenhoff TH, van der Meer JW. Innate immunity to Mycobacterium tuberculosis. Clin Microbiol Rev. 2002 4; 15(2):294–309. Review. 11932234 10.1128/CMR.15.2.294-309.2002 PMC118070

[6] Rytter MJ, Kolte L, Briend A, Friis H, Christensen VB. The immune system in children with malnutrition—a systematic review. PLoS One. 2014 8 25; 9(8):e105017 10.1371/journal.pone.0105017 25153531 PMC4143239

[7] Kashyap RS, Nayak AR, Gaherwar HM, Bhullar SS, Husain AA, Shekhawat SD, et.al Laboratory investigations on the diagnosis of tuberculosis in the malnourished tribal population of melghat, India. PLoS One. 2013 9 12; 8(9):e74652 10.1371/journal.pone.0074652 24069327 PMC3772098

[8] Ariga H, Nagai H, Kurashima A, Hoshino Y, Shoji S, Nakajima Y. Stratified Threshold Values of QuantiFERON Assay for Diagnosing Tuberculosis Infection in Immunocompromised Populations. Tuberc Res Treat. 2011; 2011:940642 10.1155/2011/940642 22567271 PMC3335708

[9] Klein S, Jeejeebhoy KN. The malnourished patient: Nutritional assessment and management In: Feldman M, Friedman LS, Sleisenger MH, editors. Sleisenger & Fordtran’s Gastrointestinal and Liver Disease. 7th ed Philadelphia: WB Saunders Co; 2002: 265–85.

[10] Kashyap RS, Rajan AN, Ramteke SS, Agrawal VS, Kelkar SS, Purohit HJ, et.al Diagnosis of tuberculosis in an Indian population by an indirect ELISA protocol based on detection of Antigen 85 complex: a prospective cohort study. BMC Infect Dis. 2007 7 10; 7:74 17620147 10.1186/1471-2334-7-74 PMC1933431

[11] Kashyap RS, Kainthla RP, Mudaliar AV, Purohit HJ, Taori GM, Daginawala HF. Cerebrospinal fluid adenosine deaminase activity: A complimentary tool in the early diagnosis of tuberculous meningitis. *Cerebrospinal Fluid Research*. 2006; 3:5. 16571142 10.1186/1743-8454-3-5 PMC1448186

[12] Jain RK, Nayak AR, Husain AA, Panchbhai MS, Chandak N, Purohit HJ, et.al Mycobacterial dormancy regulon protein Rv2623 as a novel biomarker for the diagnosis of latent and active tuberculous meningitis. Dis Markers. 2013; 35(5):311–6. 10.1155/2013/309816 24167379 PMC3787564

[13] Kashyap RS, Saha SM, Nagdev KJ, Kelkar SS, Purohit HJ, Taori GM, et.al Diagnostic markers for tuberculosis ascites: a preliminary study. Biomark Insights. 2010 8 25; 5:87–94. 20838606 10.4137/bmi.s5196 PMC2935815

[14] Husain AA, Daginawala HF, Purohit HJ, Taori GM, Kashyap RS. Assessment of immune response to culture filtrate antigens of M. tuberculosis Culture Using invitro PBMC Model: Prospects to New Vaccine development. European Journal of Experimental Biology, 2013, 3(3):35–42

[15] MacIver NJ, Michalek RD, Rathmell JC. Metabolic Regulation of T Lymphocytes. Annual review of immunology. 2013 10.1146/annurev-immunol-032712-095956 PMC3606674 23298210

[16] Schlaudecker EP, Steinhoff MC, Moore SR. Interactions of diarrhoea, pneumonia, and malnutrition in childhood: recent evidence from developing countries. Curr Opin Infect Dis. 2011; 24:496–502. 10.1097/QCO.0b013e328349287d 21734569 PMC5454480

[17] Gregor MF, Hotamisligil GS. Inflammatory mechanisms in obesity. Annual review of immunology. 2011; 29:415–445. 10.1146/annurev-immunol-031210-101322 21219177

[18] Pelletier DL, Frongillo EA Jr, Schroeder DG, Habicht JP. 1995 The effects of malnutrition on child mortality in developing countries. Bull World Health Organ. 73:443–48. 7554015 PMC2486780

[19] Matarese G. Leptin and the immune system: how nutritional status influences the immune response. Eur Cytokine Netw. 2000 3; 11(1):7–14. Review. 10705294

[20] Lord GM, Matarese G, Howard JK, Baker RJ, Bloom SR, Lechler RI. Leptin modulates the T-cell immune response and reverses starvation-induced immunosuppression. Nature. 1998 8 27; 394(6696):897–901. 9732873 10.1038/29795

[21] Friedman JM, Halaas JL. Leptin and the regulation of body weight in mammals. Nature. 1998 10 22; 395(6704):763–70. Review. 9796811 10.1038/27376

[22] Wieland CW, Florquin S, Chan ED, Leemans JC, Weijer S, Verbon A et.al Pulmonary Mycobacterium tuberculosis infection in leptin-deficient ob/ob mice. Int Immunol. 2005 11; 17(11):1399–408. 16141243 10.1093/intimm/dxh317

[23] Maffei M, Halaas J, Ravussin E, Pratley RE, Lee GH, Zhang Y et al Leptin levels in human and rodent: measurement of plasma leptin and ob RNA in obese and weight-reduced subjects. Nat Med. 1995 11; 1(11):1155–61. 7584987 10.1038/nm1195-1155

[24] Saka AO, Saka MJ, Ojuawo A, Abdulkarim A, Bilamin S, Latubosun L et. al Haematological profile in children with protein energy malnutrition in North Central Nigeria. Global Journal of Medical research. 2012 5; 12(4): 1–7.

[25] Borelli Primavera, Blatt Solange L, Rogero, Marcelo M et. al Haematological alterations in protein malnutrition. Revista Brasileira de Hematologia e Hemoterapia. 2004 3; 26(1): 49–56.

[26] Kumar GS, Venugopal AK, Mahadevan A, Renuse S, Harsha HC, Sahasrabuddhe NA, et.al Quantitative proteomics for identifying biomarkers for tuberculous meningitis. Clin Proteomics. 2012 11 30; 9(1):12 10.1186/1559-0275-9-12 23198679 PMC3572431

[27] Kashyap RS, Dobos KM, Belisle JT, Purohit HJ, Chandak NH, Taori GM, et.al Demonstration of components of antigen 85 complex in cerebrospinal fluid of tuberculous meningitis patients. Clin Diagn Lab Immunol. 2005 6;12(6):752–8 15939750 10.1128/CDLI.12.6.752-758.2005 PMC1151969

[28] Keusch GT. The history of nutrition: malnutrition, infection and immunity. J Nutr 2003; 133:336S–40S. 12514322 10.1093/jn/133.1.336S

[29] Scrimshaw NS. Historical concepts of interactions, synergism and antagonism between nutrition and infection. J Nutr 2003; 133:316S–21S 12514318 10.1093/jn/133.1.316S

[30] Pavan Kumar N, Anuradha R, Andrade BB, Suresh N, Ganesh R, Shankar J, et.al Circulating biomarkers of pulmonary and extrapulmonary tuberculosis in children. Clin Vaccine Immunol. 2013 5; 20(5):704–11. 10.1128/CVI.00038-13 23486418 PMC3647760

[31] Fu YR, Yi ZJ, Guan SZ, Zhang SY, Li M. Proteomic analysis of sputum in patients with active pulmonary tuberculosis. Clin Microbiol Infect. 2012 12; 18(12):1241–7. 10.1111/j.1469-0691.2012.03824.x 22486982

[32] Adedapo KS, Arinola OG, Ige OM, Adedapo ADA. Combination of reduced levels of serum albumin and alpha-2-macroglobulin differentiates newly diagnosed pulmonary tuberculosis patients from patients on chemotherapy. Am J Blood Res 2006; 9: 169–172.

[33] Vandevyver S, Dejager L, Vandenbroucke RE, Libert C. An acute phase protein ready to go therapeutic for sepsis. EMBO Mol Med. 2014 1; 6(1):2–3. 10.1002/emmm.201303524 24408964 PMC3936485

[34] Borth W. Alpha 2-macroglobulin, a multifunctional binding protein with targeting characteristics. FASEB Journal 1992 6 3345–3353 1281457 10.1096/fasebj.6.15.1281457

[35] Schaer DJ, Vinchi F, Ingoglia G, Tolosano E, Buehler PW. Haptoglobin, hemopexin, and related defence pathways-basic science, clinical perspectives, and drug development. Front Physiol. 2014 10 28; 5:415 10.3389/fphys.2014.00415 25389409 PMC4211382

[36] Quaye IK. Haptoglobin, inflammation and disease. Trans R Soc Trop Med Hyg. 2008 8; 102(8):735–42. 10.1016/j.trstmh.2008.04.010 18486167

[37] Banh Le, Serum proteins as markers of nutrition: What are we treating? Practical Gastroenterology. 2006 (43): 46–64.

[38] Raghu B, Sarma GR, Venkatesan P. Effect of iron on the growth and siderophore production of mycobacteria. Biochem Mol Biol Int. 1993 10; 31(2):341–8. 8275022

[39] Serafín-López J, Chacón-Salinas R, Muñoz-Cruz S, Enciso-Moreno JA, Estrada-Parra SA, Estrada-García I. The effect of iron on the expression of cytokines in macrophages infected with Mycobacterium tuberculosis. Scand J Immunol. 2004 10; 60(4):329–37. 15379857 10.1111/j.0300-9475.2004.01482.x

[40] Wilson D, Badri M, Maartens G. 2011 Performance of serum C-reactive protein as a screening test for smear-negative tuberculosis in an ambulatory high HIV prevalence population. PLoS One 6:e15248 10.1371/journal.pone.0015248 21249220 PMC3018418

[41] Wallis RS, Wang C, Doherty TM, Onyebujoh P, Vahedi M, Laang H, et.al 2010 Biomarkers for tuberculosis disease activity, cure, and relapse. Lancet Infect. Dis. 10:68–69. 10.1016/S1473-3099(10)70003-7 20113972

[42] Opolot JO, Theron AJ, Anderson R, Feldman C. Acute phase proteins and stress hormone responses in patients with newly diagnosed active pulmonary tuberculosis. Lung. 2015 2; 193(1):13–8. 10.1007/s00408-014-9680-8 25549893

[43] Gruys E, Toussaint MJM, Niewold TA, Koopmans SJ. Acute phase reaction and acute phase proteins. Journal of Zhejiang University Science B. 2005; 6(11):1045–1056. 10.1631/jzus.2005.B1045 16252337 PMC1390650

[44] Grange JM, Kardjito T, Setiabudi I. A study of acute phase reactant proteins in Indonesian patients with pulmonary tuberculosis. Tubercle 1984 3 65 (1): 23–39 6428016 10.1016/0041-3879(84)90027-8

[45] Bresnahan KA, Tanumihardjo SA. Undernutrition, the acute phase response to infection, and its effects on micronutrient status indicators. Adv Nutr. 2014 11 14; 5(6):702–11. 10.3945/an.114.006361 25398733 PMC4224207

[46] Heinrich PC, Castell JV, Andus T. Interleukin-6 and the acute phase response. Biochem J. 1990 2 1; 265(3):621–36. Review. 1689567 10.1042/bj2650621 PMC1133681

[47] Regulation of the acute phase and immune responses: interleukin-6. Ann N YAcad Sci. 1989; 557:1–583. 2472086

[48] Knolle P, Löhr H, Treichel U, Dienes HP, Lohse A, Schlaack J, et.al Parenchymal and nonparenchymal liver cells and their interaction in the local immune response. Z Gastroenterol. 1995 10; 33(10):613–20. Review. 7502557

