# Supplementary material for: Differential Levels of Alpha-2-Macroglobulin, Haptoglobin and Sero-Transferrin as Adjunct Markers for TB Diagnosis and Disease Progression in the Malnourished Tribal Population of Melghat, India
Source: PLoS One. 2015 Aug 4;10(8):e0133928. doi: 10.1371/journal.pone.0133928 (PMC4524608; doi:10.1371/journal.pone.0133928)
Supplement: S1 File — (PDF) [file pone.0133928.s001.pdf]

NUTRITIONAL ANALYSIS

Lab Number: _______________

Name:	Age:
Birth Weight:	Weight:
Children:	Height:

1.	Occupation	
2.	Monthly income of the family?	
3.	How many people in the family adult?	
4.	Which produce from the farm do you get?	
5.	How much do you store?	
6.	For what period do you get it?	
7.	What all do you buy in the weekly market?	
8.	How many kg of cereal?	
9.	Rice	
10.	Kodo	
11.	Kutki	
12.	Jawar	
13.	Wheat	
14.	How much dal do you buy?	

		kg / day	kg / week	Yes/No
15.	What vegetables do you buy every week?			
16.	How many vegetables do you buy?			
17.	Have you grown a kitchen garden?			
18.	Do you get vegetables from the garden?			
19.	How much do you get from it?			
20.	How much cooking oil do you buy from weekly market?			
21.	How much sugar do you buy from the weekly market?			
22.	How much milk do you buy?			
23.	What type of milk do you buy?			
24.	Do you have your own cow, buffalo, goat or hen?			
25.	Do you consume eggs?			
26.	How many eggs?			
27.	Do you undertake fasts?			

28.	How many fasts in a week?			
29.	How much salt is consumed?			
30.	Which cooking method boil/ fry/ roasting?			
31.	What type of cooking vessels are used?			
32.	Do you consume alcohol?			
33.	How many days?			
34.	How much?			
35.	Do you consume tabacco/ gutka / nas?			
36.	Do you consume panmasala?			
37.	Do you smoke?			
38.	Do you visit anaganwadi?			
39.	Do you get food from anaganwadi?			

Individual Nutritional Analysis

	Meal Timing	Food item	Serving size
Breakfast			
Lunch			
Evening Snacks			
Dinner			

1.	Total water intake?	
2.	Do you have regular M.C.?	
3.	For how many days with what gap?	
4.	What kind of games do you play?	

Analysis by external appearance

Height:	Age:
Weight:	Ideal body weight: Yes / No

1.	Fat and skinfold measurement	
2.	Is there a swelling on the body or water retention?	
3.	Hair - thin spared / easily pulled out	
4.	Colour of skin - black, brown	
5.	Skin - dry, rough, patches on the skin or lines on the skin when lifted does not come back to normally easily	
6.	Legs - bow shaped/curved	
7.	Eyes – bitot's spots, night blindness, pale, black circles around	
8.	Nails - broken, white moon shaped	
9.	Gums - normal / spongy/ bleeding	
10.	Lips - normal / angular stomatitis / chelosis	

11.	Face - diffuse pigmentation / moon face	
12.	Abdomen - balloon shaped	
13.	Do you wash your hands before taking any food?	
14.	Where do you go for defecation?	
15.	Is there a weight loss?	
